# Antifungal compounds from *Streptomyces* associated with attine ants also inhibit *Leishmania donovani*

**DOI:** 10.1371/journal.pntd.0007643

**Published:** 2019-08-05

**Authors:** Humberto E. Ortega, Leonardo L. G. Ferreira, Weilan G. P. Melo, Ana Ligia L. Oliveira, René F. Ramos Alvarenga, Norberto P. Lopes, Tim S. Bugni, Adriano D. Andricopulo, Mônica T. Pupo

**Affiliations:** 1 Faculdade de Ciências Farmacêuticas de Ribeirão Preto, Universidade de São Paulo, Ribeirão Preto, SP, Brazil; 2 Laboratório de Química Medicinal e Computacional, Centro de Pesquisa e Inovação em Biodiversidade e Fármacos, Instituto de Física de São Carlos, Universidade de São Paulo, São Carlos-SP, Brazil; 3 Pharmaceutical Sciences Division, University of Wisconsin-Madison, Madison, Wisconsin, United States of America; QIMR Berghofer Medical Research Institute, AUSTRALIA

## Abstract

Bacterial strains isolated from attine ants showed activity against the insect specialized fungal pathogen *Escovopsis* and also against the human protozoan parasite *Leishmania donovani*. The bioassay guided fractionation of extracts from cultures of *Streptomyces* sp. ICBG292, isolated from the exoskeleton of *Cyphomyrmex* workers, led to the isolation of Mer-A2026B (**1**), piericidin-A_1_ (**2**) and nigericin (**3**). Nigericin (**3**) presented high activity against intracellular amastigotes of *L*. *donovani* (IC_50_ 0.129 ± 0.008 μM). *Streptomyces puniceus* ICBG378, isolated from workers of *Acromyrmex rugosus rugosus*, produced dinactin (**4**) with potent anti-*L*. *donovani* activity against intracellular amastigotes (IC_50_ 0.018 ± 0.003 μM). Compounds **3** and **4** showed good selectivity indexes, 88.91 and 656.11 respectively, and were more active than positive control, miltefosine. Compounds **1**–**4** were also active against some *Escovopsis* strains. Compounds **1** and **2** were also produced by *Streptomyces* sp. ICBG233, isolated from workers of *Atta sexdens*, and detected in ants’ extracts by mass spectrometry, suggesting they are produced in the natural environment as defensive compounds involved in the symbiotic interaction.

## Author summary

Visceral leishmaniasis, caused by *Leishmania infantum* and *L*. *donovani*, is characterized by high rate mortality worldwide. Current treatments for this disease suffer from toxicity, variable efficacy, requirements for parenteral administration and length of treatment regimens. New chemical entities and development of new drugs are important to overcome the impact of this protozoan disease. Actinobacterial strains, such as *Streptomyces*, have been a source of most naturally derived antibiotics, as well as anticancer, anthelmintic, and antifungal drugs. These microorganisms also produce small molecules important in symbiotic interactions with insects, such as fungus-growing ants, fungus-growing termites, beetles and wasps against pathogens. Several novel compounds have been reported from these microorganisms with promising biological activities. In this work we show an interesting ecologic approach for drug discovery that also shows promise for the identification of antileishmanial natural products from fungus-growing ant ecosystem. Two compounds isolated from *Streptomyces* strains showed potent activity against *L*. *donovani*, higher than the positive control (miltefosine) with high selectivity indexes.

## Introduction

Leishmaniasis is designated as Neglected Tropical Diseases (NTDs) by the World Health Organization (WHO). The visceral leishmaniasis is the most serious clinical form, produced by two *Leishmania* species, *L*. *infantum* and *L*. *donovani* [1]. There are between 50–90 thousands new cases and around 20–30 thousands deaths each year due to this form of leishmaniasis [2]. The treatment of leishmaniasis is still incomplete, since available drugs are toxic and expensive, have bioavailability issues, and need to overcome parasite resistance [3]. Miltefosine, originally launched as anticancer agent [4], was the only drug approved against leishmaniasis between 1981 and 2014 [5].

Prospecting understudied sources of natural products can contribute to the discovery of new antiprotozoal pharmacophores. *Streptomyces* associated with insects have recently emerged as a prolific and underexplored source of antimicrobials [6]. In the quadripartite symbiosis in the fungus-growing ant ecosystem between three mutualists (Attine ant, fungal garden and symbiotic actinomycetes) and one parasite (specialized pathogenic fungus *Escovopsis* sp.), some interspecies interactions are mediated by small molecules [7]. The ant associated actinobacteria produce secondary metabolites to inhibit the pathogen (*Escovopsis* sp.) but not the crop fungus (phylum Basidiomycota) [8]. This specific ecological function can guide the discovery of natural products potentially active against human pathogens [8]. Indeed, interesting bacterial-derived natural products have been reported with a wide spectrum of biological activities such as dentigerumycin [9], 9-methoxyrebeccamycin [10] and selvamicin [11].

In an ongoing International Cooperative Biodiversity Group (ICBG) initiative [12], we have isolated several actinobacteria strains from the exoskeleton of fungus-growing ants to prospect for antifungal and antiprotozoal compounds. There are some examples of compounds presenting both antifungal and antiprotozoal activities, such as azoles [13] and amphotericin B [14]. Therefore, bacterial symbionts of attine ants represent an underexplored ecosystem to search for antiprotozoal natural products based on their antifungal activity against *Escovopsis* in their niches.

## Materials and methods

### General experimental procedures

RP HPLC was performed using a Shimadzu Prominence HPLC system and a Phenomenex Luna C_6_-Phenyl column (5μm, 250 x 10 mm). The mass spectrometry data for **2** and **3** were acquired with a Bruker MaXis Quadrupole Time-of-Flight MS coupled to a Waters Acquity UPLC system operated by Bruker Hystar software, and for **1** and **4** with an Accela UHPLC (Thermo Scientific, USA) apparatus with an 80 Hz photodiode array detector (PDA) coupled to a Q-Exactive Plus Orbitrap mass analyzer (Thermo Scientific, USA). NMR spectra of **1**–**4** were obtained in CDCl_3_ with a Varian Unity-Inova 500 MHz spectrometer. The LC-HRESIMS and MS/MS spectra of organic extracts of *Atta sexdens* workers were acquired with a UPLC (Shimadzu) coupled to a micrOTOF II mass spectrometer (Bruker Daltonics).

### Insect collection and isolation of actinobacteria

About 1–10 grams of the fungal gardens of the attine ants’ colony were collected. Five ants from each colony were selected for isolation of actinobacteria. Ants were identified at genus level using genera identification keys [15–17]. Specialists in taxonomy identified respective species. Ten actinobacterial strains were isolated from *Acromyrmex rugosus rugosus* worker ants, eight strains were isolated from *Cyphomyrmex* workers and twelve strains from *Atta sexdens* soldier ants. The bacterium *Streptomyces puniceus* AB10 (strain ICBG378) was isolated from *A*. *rugosus rugosus* ants collected at USP-Ribeirão Preto campus as previously described in Ortega et al. [18]. The bacterium *Streptomyces* sp. ICBG292 was isolated from the exoskeleton of *Cyphomyrmex* workers; and *Streptomyces* sp. ICBG233 from exoskeleton of *A*. *sexdens* workers. *Cyphomyrmex* and *A*. *sexdens* ants were collected in October of 2015 at the campus of the USP-Ribeirão Preto, as part of the ICBG-Brazil project [12]. Permits for collection of biological samples and research on genetic resources were issued by SISBIO (authorization 46555–6) and CNPq (010936/2014-9).

Ants collected were washed with 500 μL of sterile deionized water, vortexed for 30 s and then plated on chitin medium supplemented with the antifungals nystatin and cycloheximide (per liter: 4g chitin, 0.7g K_2_HPO_4_, 0.3g KH_2_PO_4_, 0.5g MgSO_4_·5H_2_O, 0.01g FeSO_4_·7H_2_O, 0.01g ZnSO_4_·7H_2_O, 0.01g MnCl_2_·4H_2_O, 20g of agar, 0.04 g/L nystatin, and 0.05 g/L cycloheximide). After two weeks of growth at 28°C, bacterial colonies were subcultured onto International Streptomyces Project Medium 2 (ISP-2) agar with antifungals (0.04 g/L nystatin, and 0.05 g/L cycloheximide) [19].

### Identification of actinobacteria

The DNA extraction procedure was modified from Kumar et al. [20], in which the pellet was washed in 500 μL of 10.3% sucrose, centrifuged for 1 min at 10,000 g and the supernatant discarded. Then 450 μL of TSE + lysozyme were added and incubated for 20–30 min at 37°C. After, 13 μL of proteinase K was added and incubated for another 15 min at 55°C and then 250 μL of 2% SDS, gently mixed until formation of a clear solution. Then 300 μL of phenol: chloroform pH 8.0 were added and mixed and centrifuged for 10 min at 4°C. The supernatant was transferred to another tube and 60 μL of 3M NaOAc, pH 6.0 + 700 μL of isopropanol was added. The contents were mixed until "white strings" appeared and then centrifuged for 1 min to 10,000 g, and the supernatant discarded. The pellet was washed with 70% ethanol and centrifuged again at 10,000 g for 1 min. After being left overnight to completely dry the ethanol, the DNA was resuspended in 30 μL of deionized H_2_O.

PCR amplification of the 16S rRNA gene of actinobacteria was performed using two primers: 27F (5'-AGAGTTTGATCMTGGCT-3') and 1492R (5'-TACGGYTACCTTGTTACGACTT-3') [21]. The EconoTac DNA Polymerase Kit (Lucigen, USA) was used and the final reaction volume of 15 μL contained: 8 μL Econotaq, 0.5 μL of each primer 27F and 1492R, 0.5 μL DMSO, 4.5 μL Deionized H_2_O and 1 μL DNA (10ng/μL). Amplification followed the following profile: an initial denaturation step at 94°C for 3 min followed by 32 cycles of amplification of 94°C for 30s, 60°C for 30s and 72°C for 2 minutes and a final extension step of 72°C for 5 min. The PCR product was detected by agarose gel electrophoresis and visualized by ultraviolet (UV) fluorescence after staining with ethidium bromide.

The primers 27F and 1492R were used again for the sequencing of the 16S rRNA gene. The sequencing reaction of the PCR products contained: 1.5 μL 5X buffer, 1 μL primer (10 μM), 1 μL BigDye 3.1 (Applied Biosystems), 0.5 μL DMSO, 1 μL PCR product DNA and deionized water to make up the total volume of 10 μL. The program used consisted of 95°C for 3 min, followed by 35 cycles of 96°C for 10s, 58°C for 3 min and a final extent of 72°C for 7 min. The sequencing reaction was purified with the Axyprep Mag Dyeclean purification kit (Axygen) in which 5 μL of magnetic beads solution and 31 μL of 85% ethanol were added for each reaction. The tubes were placed on a magnetic plate for 3 min and then the liquid was removed. 100 μL of 85% ethanol was added for 30s and then the liquid was discarded. 100 μL of 85% ethanol was added again for 30s and after discarded. The liquid was removed as much as possible with a pipette and left overnight to completely dry the ethanol. The DNA was resuspended in 25 μL of deionized H_2_O.

Sequencing was performed at the Center for Genetics and Biotechnology at the University of Wisconsin—Madison (Biotech Center, UW—Madison, WI, USA). The sequences were edited and used for assembly of the contigs in the SecMan Pro Software (DNASTAR). Contigs were used to search for homologous sequences in the NCBI—GenBank (https://blast.ncbi.nlm.nih.gov/Blast.cgi) and Eztaxon (http://www.ezbiocloud.net/eztaxon/identify). The sequences are deposited at NCBI GenBank under Accession numbers: MK118901 (ICBG233) and MK118902 (ICBG292).

### *In vitro* evaluation of natural products on *L*. *donovani* promastigotes, intracellular amastigotes and human macrophages

*Leishmania donovani* axenic cultures (strain MHOM/ET/67/HU3) were maintained in M199 medium (pH 7.4) supplemented with 10% heat-inactivated fetal calf serum (FCS) and grown at 28°C [22]. Human leukemia cells (THP-1 cell line) were maintained in RPMI-1640 (FCS 10%) and grown at 37°C and 5% CO_2_. Stock solutions of compounds **1**–**4** were prepared in 100% DMSO at 10 mM and tested in 2-fold serial dilutions (10 concentrations) in 96-well flat-bottom microtiter plates.

For the promastigote assay, *L*. *donovani* cells from axenic cultures in logarithmic growth were seeded at 1 x 10^5^/well (M199, 80 μL) and compounds were added in serial dilutions (20 μL). All plates included negative controls (100% parasite growth) and miltefosine as a positive control. After 72 hours of incubation at 28°C, 10 μL of Alamar Blue (12.5 mg resazurin/100 mL distilled water) [23] was added to each well and then the plates were incubated for 3 hours. This indicator of cell viability permeates into viable parasites, where it is reduced by NADPH and NADH enzymes to the highly fluorescent compound resorufin [24]. Following incubation, the plates were read with a microplate fluorometer under an excitation wave length of 536 nm and an emission wave length of 588 nm. If the test compound is inactive against the *L*. *donovani*, parasite remains viable and it is able to convert resazurin into resorufin, resulting in fluorescence emission. If the test compound is active against *L*. *donovani*, the number of viable parasites is reduced, thus resulting a decrease in fluorescence [25]. Growth inhibition was expressed as a percentage of the fluorescence of the negative control wells. IC_50_ values were determined using SigmaPlot. Dose-response curves were fitted using log (inhibitor concentration) vs. normalized response (between 0% and 100%) with variable slope, and IC_50_ values were automatically calculated.

In the intracellular amastigote assay, THP-1 cells were seeded at 2 × 10^4^/well (RPMI-1640, 100 μL) with phorbol 12-myristate 13-acetate (PMA) at 20 ng/mL for differentiation into macrophages. After incubation for 72 hours (5% CO_2_, 37°C), medium was aspirated and late-stage promastigotes were added (2 × 10^5^/well, 100 μL). After 24 hours of incubation, medium was aspirated to clear extracellular parasites, compounds were added in serial dilutions (100 μL) and the plates were incubated for 120 hours. All plates included negative controls (100% parasite growth) and miltefosine as a positive control. Following incubation, medium was removed and the cells were fixed in methanol and stained with Giemsa. The average number of intracellular amastigotes per THP-1 cell was determined using an inverted microscope and a cell counter [26]. Growth inhibition was expressed as a percentage of the average number of amastigotes per macrophage in the negative control wells. IC_50_ values were determined as described above for the promastigote assay.

For the selectivity assay, THP-1 cells were seeded at 2 × 10^4^/well (RPMI-1640, 100 μL) with PMA at 20 ng/mL for differentiation into macrophages [27]. After incubation for 72 hours (5% CO_2_, 37°C), medium was aspirated, compounds were added in serial dilutions (100 μL) and the plates were incubated for 120 h. All plates included negative controls and doxorubicin as a positive control. Following incubation, 10 μL of Alamar Blue was added to each well and then the plates were incubated for 3 hours. Next, the plates were read with a microplate fluorometer under an excitation wave length of 536 nm and an emission wave length of 588 nm. Growth inhibition was expressed as a percentage of the fluorescence of the negative control wells. IC_50_ values were determined as described above for the promastigote and amastigote assays.

### Isolation of compounds 1–3

Seed cultures of *Streptomyces* sp. ICBG292 were initially grown in 40 mL of ISP-2 (4 tubes of 25 × 150 mm) in a shaker for 7 days at 28°C and 200 rpm. The bacterium was inoculated into ISP-2 broth (4 g yeast extract, 10 g malt extract, and 4 g glucose per liter) in a Fernbach flask (1 L of medium in a flask of 2.8 L + 70 g of HP20) for 7 days at 28°C and 200 rpm. The HP20 and cells were filtered and washed with water and extracted with acetone (2 L). The organic solvent was filtered and dried under vacuum. A liquid-liquid partition using ethyl acetate/water was carried out, the organic phase was separated and dried to give the crude organic extract (271.7 mg). The extract was purified by SPE-C18 (55 μm, 1 g) using the following gradient: 10 mL (20% MeOH-H_2_O, **A1:** 55.1 mg); 10 mL (40% MeOH-H_2_O, **A2:** 23.5 mg); 10 mL (60% MeOH-H_2_O, **A3:** 14.5 mg); 10 mL (80% MeOH-H_2_O, **A4:** 47.2 mg); and 10 mL (100% MeOH, **A5:** 85.5 mg). Fractions **A4** and **A5** were active against *Escovopsis* (**S13 Fig**), so they were combined and further purified by semi-preparative HPLC using the column C_6_-Phenyl (5μm, 250 x 10 mm) and the following gradient at 4 mL/min: 1–20 min, linear gradient from 70% MeOH-H_2_O to 100% MeOH; 20–25 min, isocratic flow of 100% MeOH; 25–25.5 min, linear gradient from 100% MeOH to 70% MeOH-H_2_O; 25.5–30.5 min, isocratic flow of 70% MeOH-H_2_O to give 13 fractions [**A5.1** (2.5 mg); **A5.2** (5.0 mg); **A5.3** (3.1 mg); **A5.4** (2.6 mg); **A5.5** (3.1 mg); **A5.6** (15.5 mg), **A5.7** (10.9 mg), **A5.8** (2.2 mg), **A5.9** (14.3 mg), **A5.10** (1.6 mg), **A5.11** (0.9 mg), **A5.12** (1.2 mg), **A5.13** (4.3 mg)]. Fractions **A5.2**, **A5.6** and **A5.9** were identified by NMR and HRESIMS as antibiotics Mer-A2026B (**1**), piericidin-A_1_ (**2**) and nigericin (**3**), respectively (**S1–S6 Figs**). Purity of compounds was measured by HPLC as 99% for compound **1**, 97% for compound **2** and 93% for compound **3**.

### Isolation of compound 4

Seed culture of *S*. *puniceus* ICBG378 was initially grown in 10 mL of ISP-2 (25 × 150 mm tube) and was mounted into a shaker for 2 days at 28°C and 200 rpm. The bacterium was inoculated in broth A-medium (20 g soluble starch, 10 g glucose, 5 g peptone, 5 g yeast extract, 5 g CaCO_3_ per liter) in a baffled Erlenmeyer flask [2 x (100 mL of medium in a flask of 500 mL + 4 mL of seed culture + 7 g of HP20)] for 7 days at 28°C and 200 rpm. The HP20 was filtered and washed with distilled water and acetone. The organic solvent was filtered and dried under vacuum to give the crude extract (235.68 mg), which was purified by SPE-ENV+ (55 μm, 1 g) using the following gradient: 10 mL (25% MeOH-H_2_O, **B1:** 50.1 mg); 10 mL (50% MeOH-H_2_O, **B2:** 20.5 mg); 10 mL (75% MeOH-H_2_O, **B3:** 41.3 mg); 10 mL (100% MeOH, **B4:** 102.5 mg). Fractions **B3** and **B4** were active against *Escovopsis* (**S13 Fig**). They were mixed and purified by SPE-Si (55 μm, 500 mg) with the gradient: 8 mL [100% Hexane, **B4.1**: 32.6 mg], 8 mL [Hexane:EtOAc (8:2), **B4.2**: 57.0 mg], 8 mL [Hexane:EtOAc (6:4), **B4.3**: 8.9 mg], [Hexane:EtOAc (4:6), **B4.4**: 6.7 mg], 8 mL [Hexane:EtOAc (2:8), **B4.5**: 3.5 mg], 8 mL [100% EtOAc, **B4.6**: 2.9 mg], and 8 mL [100% Methanol, **B4.7**: 15.3 mg]. The fraction **B4.2** was identified by NMR and HRESIMS as dinactin (**4**) with 91% purity as measured by HPLC (**S7**and **S8 Figs**).

### Antagonist bioassay of bacterial strains and compounds against fungi

Each bacterium-fungus and compound-fungal challenge was replicated two times on ISP-2 agar. Bacteria strains were initially screened against *Escovopsis* sp. ICBG1251. Bacteria were placed in the center of ISP-2 agar Petri dishes and grown alone during 7 days; fungal strains were then point-inoculated near the edge of the culture (microbial strains distant from each other around 3 cm). Two microliters of compounds (100 μg) were placed in the center of Petri dishes and fungal strains were then point-inoculated near the edge of the plate. The positive control used was the miconazole. Challenges were monitored each 7 days and inhibition zone was measure after 21 days [8]. Four different fungal strains were used for testing the pure compounds: *Escovopsis* sp. ICBG711 (from *Trachymyrmex* colony), *Escovopsis* sp. ICBG740 (from *Acromyrmex* colony), *Escovopsis* sp. ICBG1251 (from *Atta* colony) and *Trichoderma* sp. ICBG1100 (from attine colony).

### Identification of compounds 1 and 2 from *Atta sexdens* exoskeleton

*Atta sexdens* colonies, collected at USP-Ribeirão Preto campus, were kept under laboratorial conditions. A total of 25 *A*. *sexdens* individuals, obtained from these colonies, were mechanically cleaned using small forceps and extracted with 50 mL of methanol. The extracts were filtered and evaporated to dryness. The crude extracts were evaluated for the presence or absence of compounds **1** and **2** by LC-HRESIMS, as described at general procedures.

## Results and discussion

Ten bacterial strains were isolated from *A*. *rugosus rugosus* ants, eight strains were recovered from *Cyphomyrmex* ants and twelve from *A*. *sexdens*. All 30 bacterial strains were challenged in antagonism assays against *Escovopsis* sp., the specialized pathogenic fungus of Attine ants, and bioactive strains were identified through 16S rRNA sequencing. *Streptomyces puniceus* ICBG378 from *A*. *rugosus rugosus*, *Streptomyces* sp. ICBG292 from *Cyphomyrmex* sp., and *Streptomyces* sp. ICBG233 from *A*. *sexdens* showed high inhibition of *Escovopsis*, and were selected for scale up culturing and antiprotozoal assays. Crude extracts and fractions of cultures of the three selected *Streptomyces* strains inhibited the growth of *L*. *donovani* promastigotes (inhibition higher than 90%). Therefore, they were selected for the isolation and characterization of biologically active natural products.

**Fig 1:**
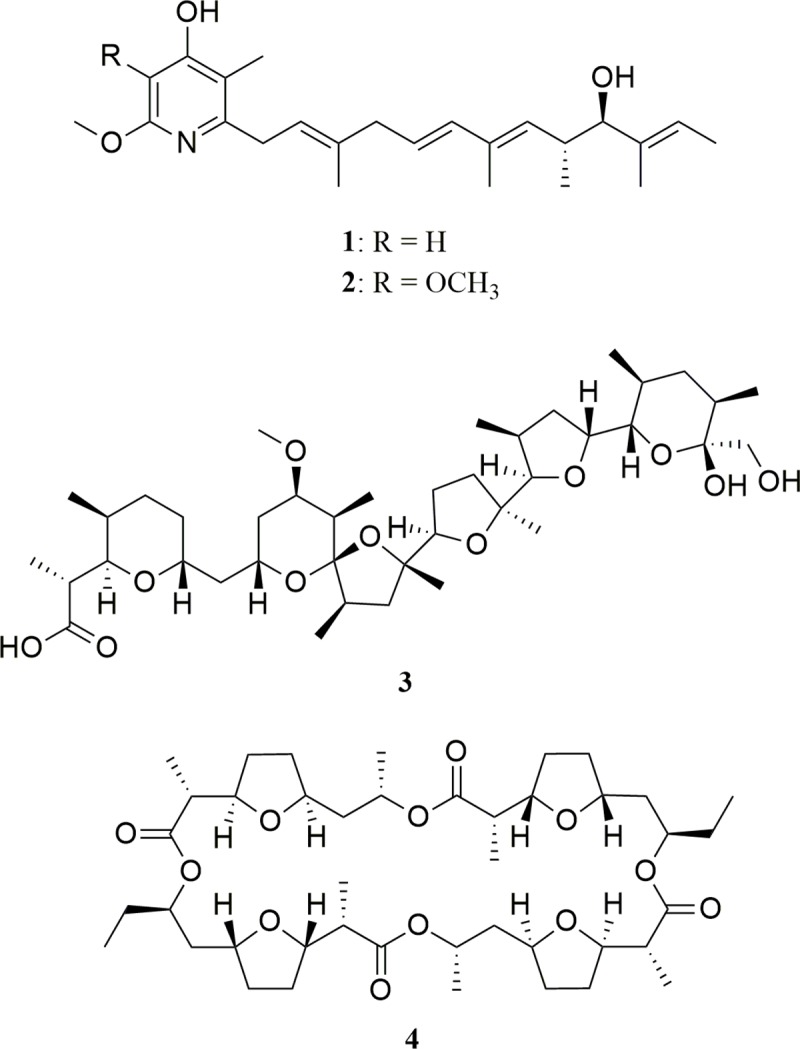
Compounds identified from bacteria *Streptomyces* sp. ICBG292 (1–3), *Streptomyces* sp. ICBG233 (1, 2), and *S*. *puniceus* ICBG378 (4).

The fractionation of extracts was guided by the antifungal assay against *Escovopsis* (**S1 Fig**) and led to the isolation of the known antibiotics mer-A2026B [28] (**1**), piericidin-A_1_ [29] (**2**), nigericin [30,31] (**3**), produced by *Streptomyces* sp. ICBG292 (**Fig 1**); and dinactin [32] (**4**), produced by *S*. *puniceus* ICBG378. Compounds **1** and **2** were also isolated from *Streptomyces* sp. ICBG233, associated with *A*. *sexdens* ants. Structures were established on the basis of NMR and HRESIMS data and comparison with literature (**S2–S14 Figs**).

**Fig 2:**
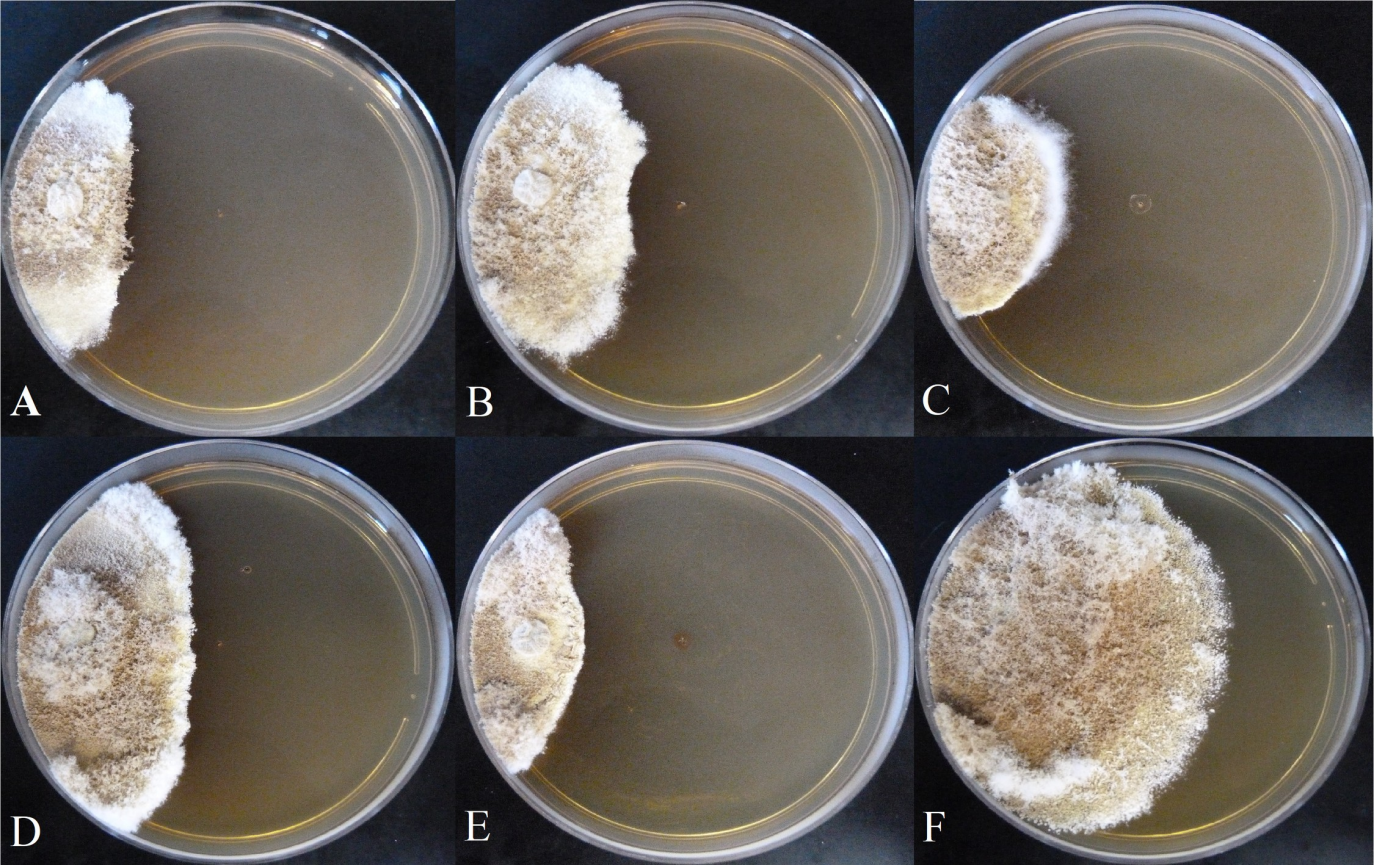
ICBG741 (21 days of growth at 28°C). **Antagonist activity of compounds (100 μg) against *Escovopsis* sp. A)** Mer-A2026B (**1**), **B)** piericidin-A_1_ (**2**), **C)** nigericin (**3**), **D)** dinactin (**4**), **E)** miconazole, and **F)** negative control.

**Fig 3:**
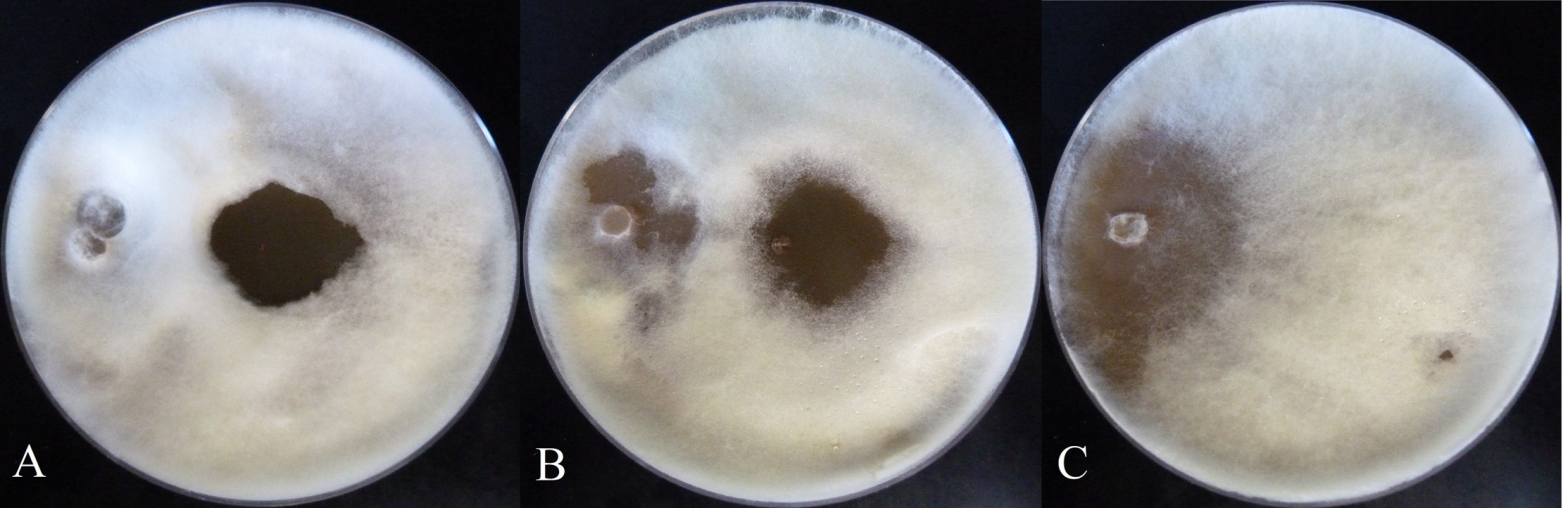
Antagonist activity of compounds (100 μg) against *Trichoderma* sp ICBG1100 (21 days of growth at 28°C). **A)** Mer-A2026B (**1**), **B)** Miconazole, **C)** Negative control.

Compounds **1**–**4** were active against *Escovopsis* sp. ICBG740 (**Fig 2**). Compound **1** showed higher antagonist activity against four different *Escovopsis* strains compared to compounds **2**–**4** (**Fig 2**and **S15**–**S17 Figs**), with inhibition zone similar to the positive control (miconazole). Compound **1** was also active against the fungus *Trichoderma* sp. (**Fig 3**).

All compounds were active against *L*. *donovani* promastigotes while compounds 1, 2 and 4 were also active against intracellular amastigotes (**Table 1**). This is the first report of the antileishmanial activity of antibiotics Mer-A2026B (**1**), piericidin-A_1_ (**2**) and dinactin (**4**). *L*. *donovani* lives in the sandfly gut as promastigotes. Promastigotes are the infective stage of *Leishmania* sp., being transmitted to humans via the bite of sandflies. Skin macrophages phagocyte the promastigotes, where the promastigotes differentiate into amastigote form. Intracellular amastigotes reproduce within the macrophages, eventually rupturing the host cell to infect other surrounding macrophages [33]. In addition to being involved in different stages of the life cycle of the parasite, promastigotes and amastigotes differ morphologically. Promastigotes are flagellated elongated cells, while amastigotes are rounded non-flagellated cells [26]. Compounds **3** and **4** were more active against both *L*. *donovani* forms than the positive control miltefosine (**Table 1**). Although intracellular amastigotes are the clinically relevant form, assessing the activity against both parasite stages can provide important information for further studies on the mechanism of action of these compounds. These activity data can be useful to investigate which biochemical pathways are modulated and understand the role played by the respective molecular targets in each stage of the parasite life cycle. The selectivity index, which is the ratio between the activity against THP-1 macrophages and intracellular amastigotes, indicates whether the compounds are selective for *L*. *donovani* over the human host cells. The probability of a compound to elicit cytotoxic effects in the human host decreases as the selectivity index increases. Therefore, the selectivity index is an important safety metric, and was assessed for compounds **3** and **4**. The selectivity indexes of **3** and **4** were 88.91 and 656.11, respectively, suggesting their safety.

**Table 1:** Activity of compounds 1–4 on *L*. *donovani* intracellular amastigotes, against promastigotes and THP-1.

Compounds	IC_50_ (μM) Intracellular amastigotes	IC_50_ (μM) Promastigotes	CC_50_ (μM) THP-1^a^	Selectivity Index^b^
**1**	49.85 ± 7.01	35.86 ± 1.83	> 64	—
**2**	> 64	38.41 ± 4.63	> 64	—
**3**	0.129 ± 0.008	0.284 ± 0.072	11.47 ± 0.68	88.91
**4**	0.018 ± 0.003	0.032 ± 0.005	11.81 ± 1.57	656.11
Doxorubicin	—	—	0.571 ± 0.068	—
Miltefosine	5.80 ± 0.59	4.74 ± 0.25	—	—

Data are shown as mean ± SD (*n* = 2 biological replicates)

^a^THP-1 human leukemia macrophages (host cells of *L*. *donovani*)

^b^Selectivity index = CC_50_ THP-1/IC_50_ intracellular amastigotes

A potent vasodilating activity has been reported for mer-A2026B (**1**) [34]; and insecticidal, antimicrobial and cytotoxic activities for piericidin-A_1_ (**2**) [35–37]; while strong antibacterial and anticancer activities have been found for nigericin (**3**) and dinactin (**4**) [38–40]. The high activities and good selectivity indexes obtained for nigericin (**3**) and dinactin (**4**) in our experiments (**Table 1**) are in agreement with previous data for nigericin monosodium salt and nonactin, an analogue of **4**, using *ex-vivo* splenic explant culture system from hamsters infected with *L*. *donovani* [41].

Compounds **3** and **4** are considered ionophores that reversibly bind and transport ions across biological membranes [42]. Nigericin (**3**) has been shown to move sodium and potassium ions through membranes [43]. When bound to a cation, nigericin loses a proton and generates an uncharged species that can permeate into cell membranes, acting as a carrier. The molecule can also permeate into membranes as a protonated noncomplexed molecule. Nigericin can promote an exchange of K^+^ for H^+^ that results in the modification of the ion gradient across the membranes involved in the energetic metabolism [44]. Dinactin (**4**) is one member of the family of macrotetrolide nactins with ability to selectively complex a wide variety of cations [45]. Few ionophore compounds have been described to inhibit *L*. *donovani*. One example is the ionophore A23187 that binds Ca^2+^ and kills intracellular *Leishmania* in the presence of lipopolysaccharide (LPS), mediated by generation of L-arginine-dependent nitrogen oxidation products [46]. Another ionophore, named calcimycin, has been described to kill *Leishmania* promastigotes by activating parasite nitric oxide synthase [47]. The *Leishmania* cell death is accompanied by the loss of mitochondrial polarization and plasma membrane integrity and can be blocked by specific inhibitors of constitutive Ca^2+^/calmodulin-dependent nitric oxide synthase [47]. The most recognized mechanism of action of miltefosine against *L*. *donovani* is the inhibition of phospholipid synthesis and cytochrome *c* oxidase, but recently another mechanism has been described based in the abrupt increase in the intracellular Ca^2+^ concentration in the *L*. *donovani* [48], a similar property of ionophores.

*L*. *donovani* lives in the sandfly gut as promastigotes and in mammalian macrophages as amastigotes [49]. This protozoan extrudes protons through H^+^-ATPase to regulate intracellular pH and to facilitate nutrient uptake [49]. This proton extrusion is enhanced by the addition of K^+^ [49]. This could be one mechanism by which nigericin controls the growth of *Leishmania* parasite. The mechanisms of action of nigericin (**3**) and dinactin (**4**) against *L*. *donovani* have not been described.

Compounds **1** and **2** showed drug-like properties according to several rules such as Lipinski and Veber filters [50,51], while **3** and **4** exceed the ideal molecular weight and number of hydrogen-bond acceptors (HBA) (**Table 2**). The computational predictions were run using SwissADME [52] and Stardrop (Optibrium) [53].

**Table 2:** Molecular properties of compound 1–4.

Code	MW	LogP	hERGpIC_50_	HIA	HBD	HBA	TPSA	nrotb	Drug-likeness	PAINS alert
**1**	385.54	4.10	5.93	+	2	4	62.58	9	yes	0
**2**	415.56	3.93	5.93	+	2	5	71.81	10	yes	0
**3**	724.96	3.77	3.96	-	3	11	142.37	9	no	0
**4**	764.98	4.70	4.43	+	0	12	142.10	2	no	0
**Desired Value**	≤ 500	< 5	< 6.3	+	≤ 5	≤ 10	≤ 140 Å	≤ 10		

LogP: octanol/water partition coefficient; hERG pIC_50_: -logIC_50_ on human ether-a-go-go-related gene potassium ion channels; HIA: human intestinal absorption; HBD: hydrogen-bond donors; HBA: hydrogen-bond acceptors; TPSA: topological surface area; nrotb: number of rotatable bonds; PAINS: pan-assay interference compounds. Drug-likeness according to Lipinski and Veber filters.

Lipinski’s rule of five states that compounds showing more than 5 hydrogen-bond donors, 10 hydrogen-bond acceptors, molecular weight greater than 500 and LogP greater than 5, are likely to show poor gastrointestinal absorption [50]. However, several natural products that do not comply with Lipinski´s rules have been approved as drugs, such as paclitaxel, rapamycin, cyclosporine A, and others. In general, natural products are considered as exceptions to Lipinski´s rules. However, the properties LogP and hydrogen-bond donors are very important for predicting bioavailability. A possible explanation is that nature can maintain low hydrophobicity and intermolecular hydrogen-bond donating potential when it needs to produce active compounds with high molecular weight and rotatable bonds; and natural products could also take advantage of active transport mechanisms since they contain biosynthetic moieties that resemble endogenous metabolites [54]. So, dinactin (**4**) could be an interesting compound for further pharmacological studies in the treatment of leishmaniasis based on the high selectivity index against *L*. *donovani* and on LogP and HBD values that comply with Lipinski´s rules. Furthermore, given their remarkable *in vitro* activity, compounds **3** and **4** are suitable starting points for molecular optimization aiming to pursue molecules that fit into the drug-like concept.

Compounds **1**–**4** can join the chemical cocktail used by actinobacteria to control the growth of the pathogenic fungus *Escovopsis* sp. and other opportunistic fungi such as *Trichoderma* sp. in fungus-growing ant colonies. Compounds **1** and **2** were also identified from *Streptomyces* sp. ICBG233 associated to workers of *Atta sexdens* and from the organic extract of these ants by mass spectrometry (**S18–S23 Figs**), confirming their production in the natural environment. Compound **2** and other piericidin derivatives together with nigericin (**3**) have also been reported from *Candidatus Streptomyces philanthi* symbiont of solitary beewolf digger wasps (*Philanthus triangulum*, Hymenoptera, Crabronidae) as antibiotic protectors of their larval offspring against pathogens [55,56]. Authors argue that the mixture of these antibiotics could help in the evolutionary stable defense against different pathogens [55,56], and the current identification of the same compounds in bacterial symbionts of attine ants reinforces this hypothesis.

Considering the remarkable activity against *L*. *donovani* shown by the identified compounds and that the treatment for visceral leishmaniasis suffers from several drawbacks, the results reported herein can contribute to the development of novel therapeutic agents for this NTD. Moreover, most current drug development approaches are based on high-throughput screening (HTS) of synthetic compound collections. HTS platforms can screen libraries containing thousands of molecules, whose chemical diversity are provided by methods such as combinatorial chemistry. Natural products can provide further structural diversity and novel chemotypes that differ from those obtained via combinatorial chemistry. In this context, natural products are a rich source of structural diversity that offers unique chemical matter to be used as reference for the design of novel leishmanicidal agents. Our results also validate the ecological approach of screening antifungal natural products from actinobacteria associated to attine ants as a good strategy for discovering antileishmanial compounds.

## Supporting information

S1 Fig Antagonist activity of fractions (2 μL of 50 μg) A4 and A5 (Containing compounds 1–3) and B3+B4 (Containing compound 4) against *Escovopsis*. (PDF)

S2 Fig ^1^H NMR of mer-A2026B (1) (CDCl_3_, 500 MHz). (PDF)

S3 Fig HRESIMS of mer-A2026B (1). (PDF)

S4 Fig ^1^H NMR of piericidin-A1 (2) (CDCl_3_, 500 MHz). (PDF)

S5 Fig ^13^C NMR of piericidin-A1 (2) (CDCl_3_, 125 MHz). (PDF)

S6 Fig HRESIMS of piericidin-A1 (2). (PDF)

S7 Fig ^1^H NMR of nigericin (3) (CDCl_3_, 500 MHz). (PDF)

S8 Fig ^13^C NMR of nigericin (3) (CDCl_3_, 125 MHz). (PDF)

S9 Fig ^13^C NMR of nigericin (3) (CDCl_3_, 125 MHz). Region of 10 to 46 ppm.
(PDF)

S10 Fig ^13^C NMR of nigericin (3) (CDCl_3_, 125 MHz). Region of 56 to 110 ppm.
(PDF)

S11 Fig HRESIMS of nigericin (3). (PDF)

S12 Fig ^1^H NMR of dinactin (4) (CDCl_3_, 500 MHz). (PDF)

S13 Fig ^13^C NMR of dinactin (4) (CDCl_3_, 125 MHz). (PDF)

S14 Fig HRESIMS of dinactin (4). (PDF)

S15 Fig Antagonist activity of compounds (100 μg) against *Escovopsis* sp ICBG711. (PDF)

S16 Fig Antagonist activity of compounds (100 μg) against *Escovopsis* sp ICBG740. (PDF)

S17 Fig Antagonist activity of compounds (100 μg) against *Escovopsis* sp ICBG1251. (PDF)

S18 Fig LC-HRESIMS of *Atta sexdens* methanolic extract (MRM *m/z* 386.40, positive mode). (PDF)

S19 Fig MS/MS spectra of compound eluted at 32.7 minutes (*m/z* 386.40), identified as mer-A2026B (positive mode). (PDF)

S20 Fig MS/MS spectra of mer-A2026B (positive mode). (PDF)

S21 Fig LC-HRESIMS of *Atta sexdens* methanolic extract (MRM *m/z* 416.30, positive mode). (PDF)

S22 Fig MS/MS spectra of compound eluted at 40.1 minutes (*m/z* 416.30), identified as piericidin A (positive mode). (PDF)

S23 Fig MS/MS spectra of piericidin A (positive mode). (PDF)
