# Supplementary material for: Antifungal compounds from Streptomyces associated with attine ants also inhibit Leishmania donovani
Source: PLoS Negl Trop Dis. 2019 Aug 5;13(8):e0007643. doi: 10.1371/journal.pntd.0007643 (PMC6695191; doi:10.1371/journal.pntd.0007643)

S1 Fig. Antagonist activity of fractions (2 µL of 50 µg) **A4** and **A5** (Containing compounds **1-3**) and **B3+B4** (Containing compound **4**) against *Escovopsis*

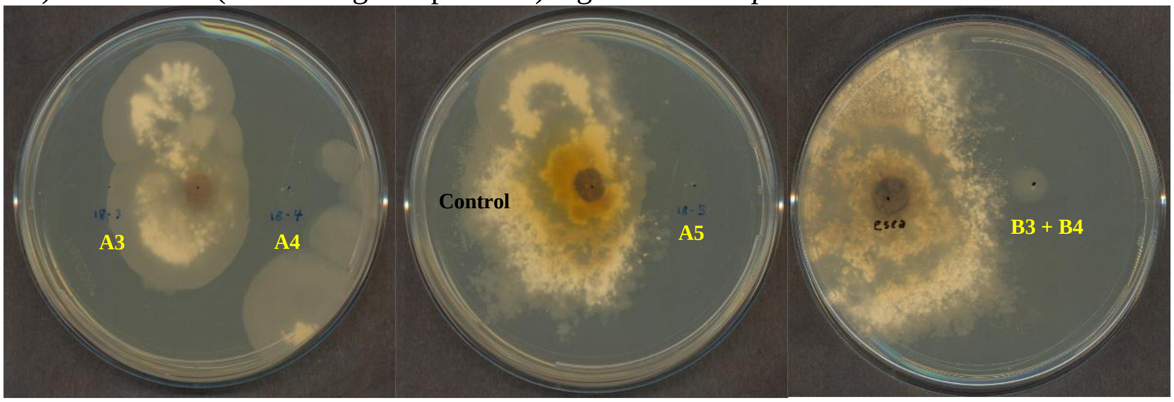

Supplement: S1 Fig — (PDF) [file pntd.0007643.s001.pdf]
