# Supplementary figures and images for: Antifungal compounds from Streptomyces associated with attine ants also inhibit Leishmania donovani
Source: PLoS Negl Trop Dis. 2019 Aug 5;13(8):e0007643. doi: 10.1371/journal.pntd.0007643 (PMC6695191; doi:10.1371/journal.pntd.0007643)

**S2 Fig.**  $^1\text{H}$  NMR of mer-A2026B (1) ( $\text{CDCl}_3$ , 500 MHz)

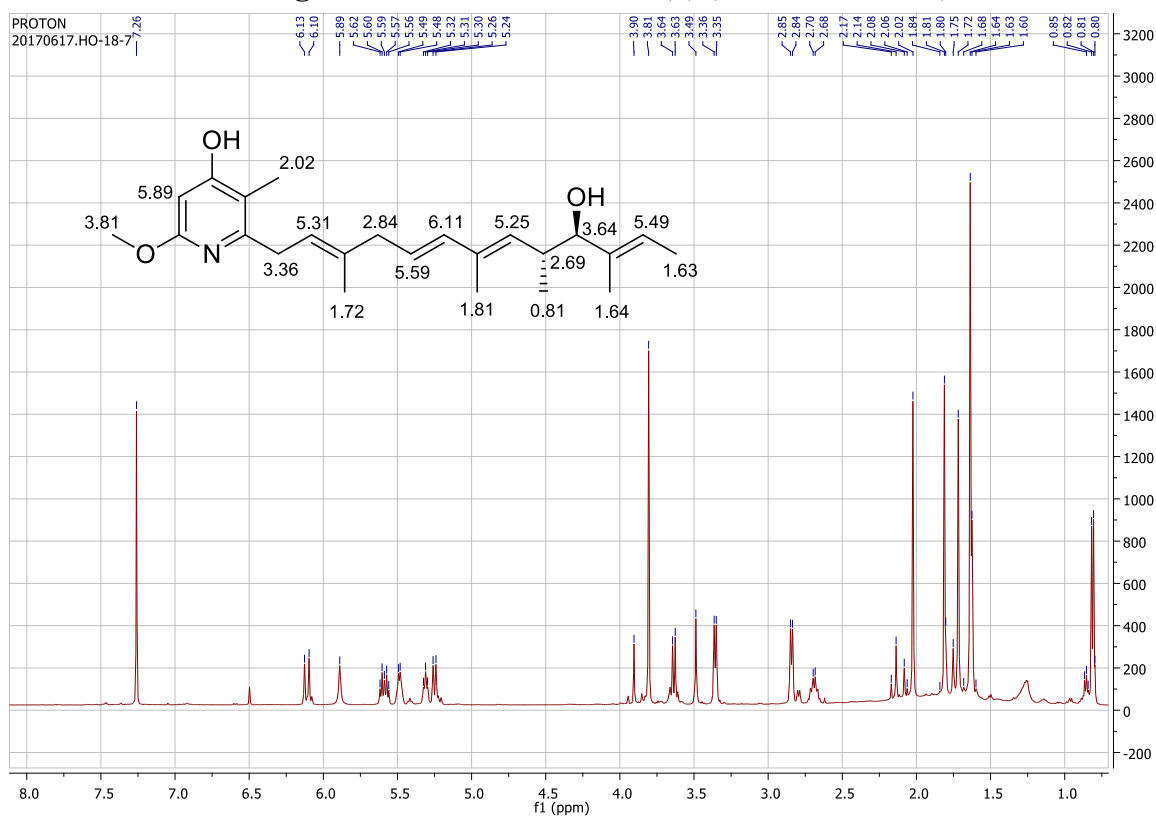

Supplement: S2 Fig — (PDF) [file pntd.0007643.s002.pdf]

**S4 Fig.**  $^1\text{H}$  NMR of piericidin-A<sub>1</sub> (**2**) ( $\text{CDCl}_3$ , 500 MHz)

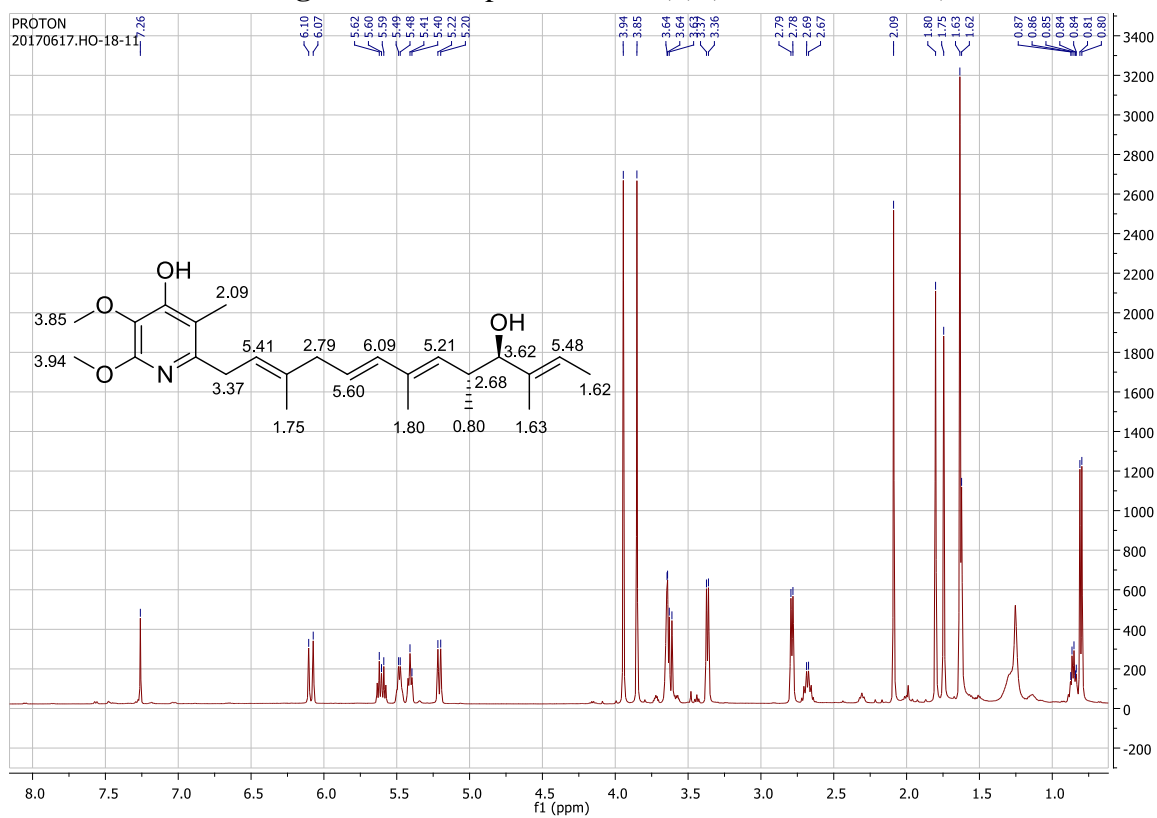

Supplement: S4 Fig — (PDF) [file pntd.0007643.s004.pdf]

**S5 Fig.**  $^{13}\text{C}$  NMR of piericidin-A<sub>1</sub> (**2**) ( $\text{CDCl}_3$ , 125 MHz)

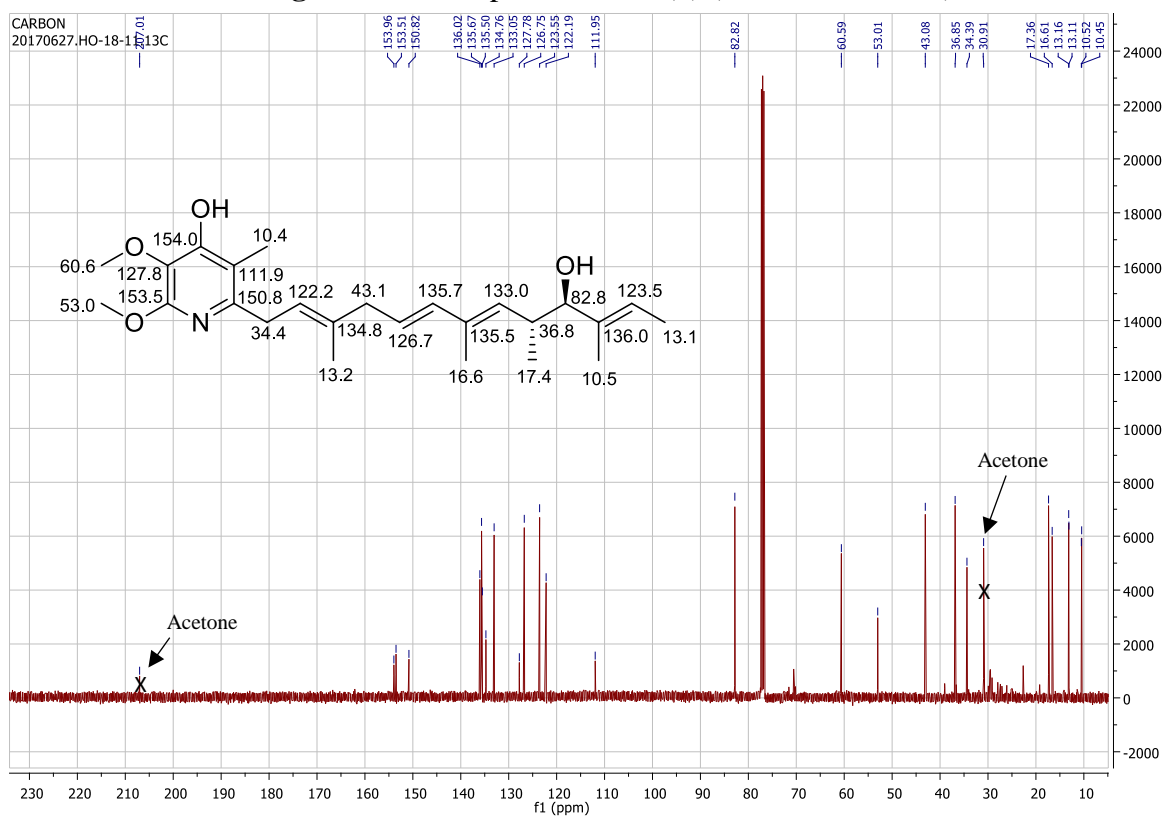

Supplement: S5 Fig — (PDF) [file pntd.0007643.s005.pdf]

**S6 Fig.** HRESIMS of piericidin-A1 (2)

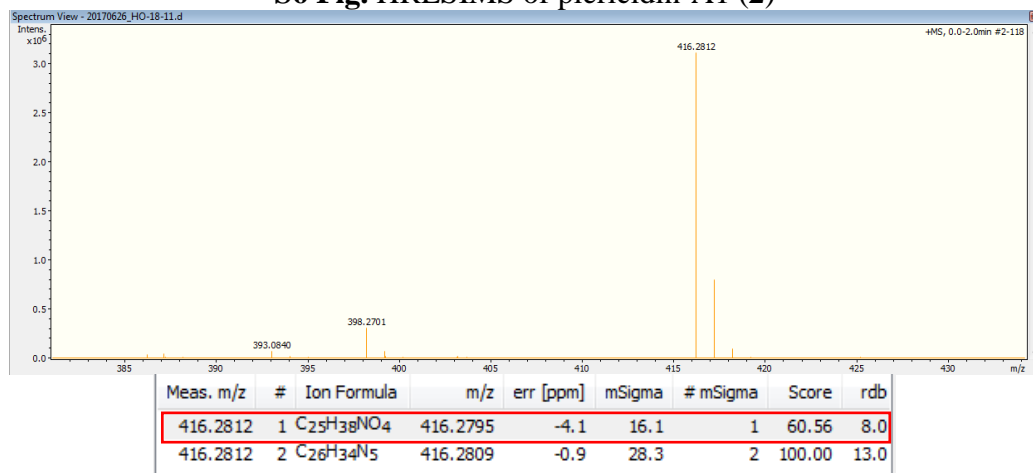

Supplement: S6 Fig — (PDF) [file pntd.0007643.s006.pdf]

**S8 Fig.**  $^{13}\text{C}$  NMR of nigericin (**3**) ( $\text{CDCl}_3$ , 125 MHz)

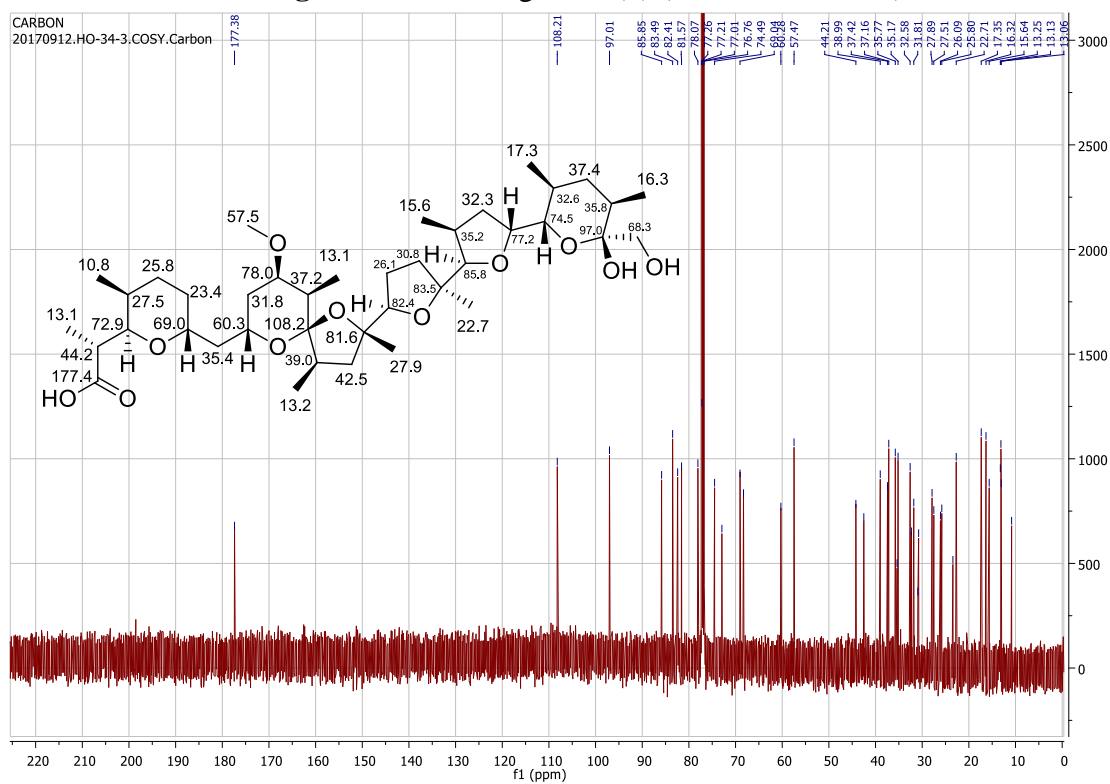

Supplement: S8 Fig — (PDF) [file pntd.0007643.s008.pdf]

**S9 Fig.**  $^{13}\text{C}$  NMR of nigericin (**3**) ( $\text{CDCl}_3$ , 125 MHz). Region of 10 to 46 ppm.

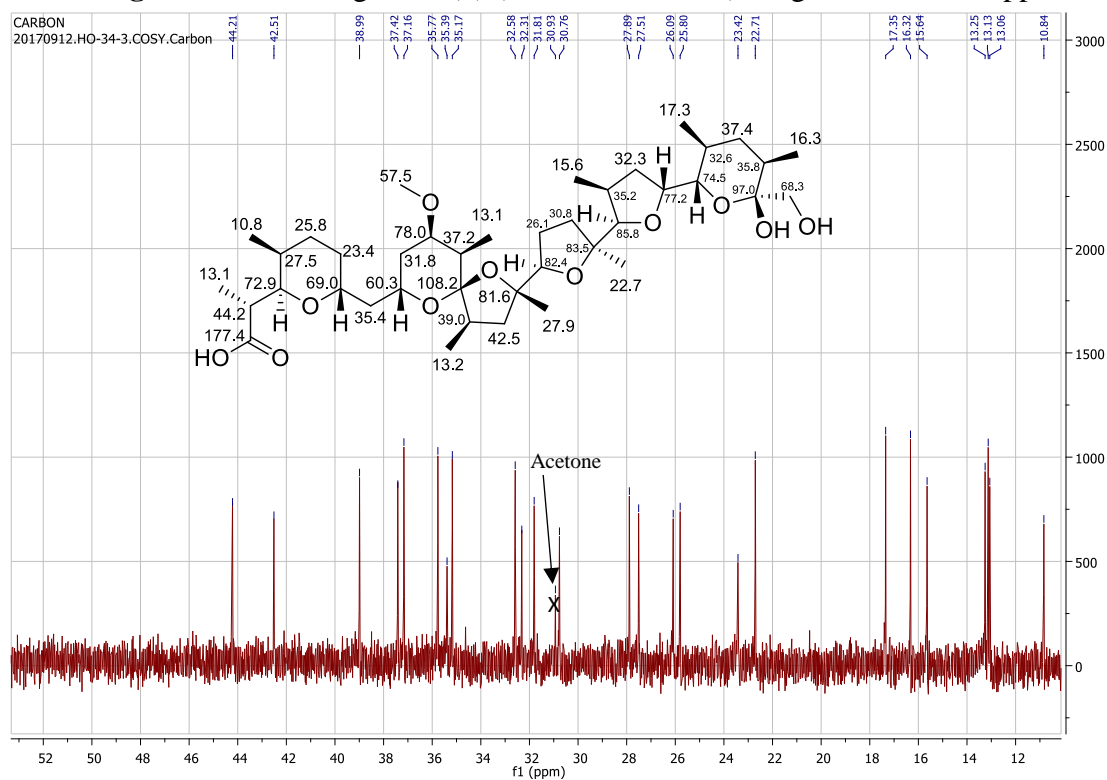

Supplement: S9 Fig — Region of 10 to 46 ppm. (PDF) [file pntd.0007643.s009.pdf]

**S10 Fig.**  $^{13}\text{C}$  NMR of nigericin (**3**) ( $\text{CDCl}_3$ , 125 MHz). Region of 56 to 110 ppm.

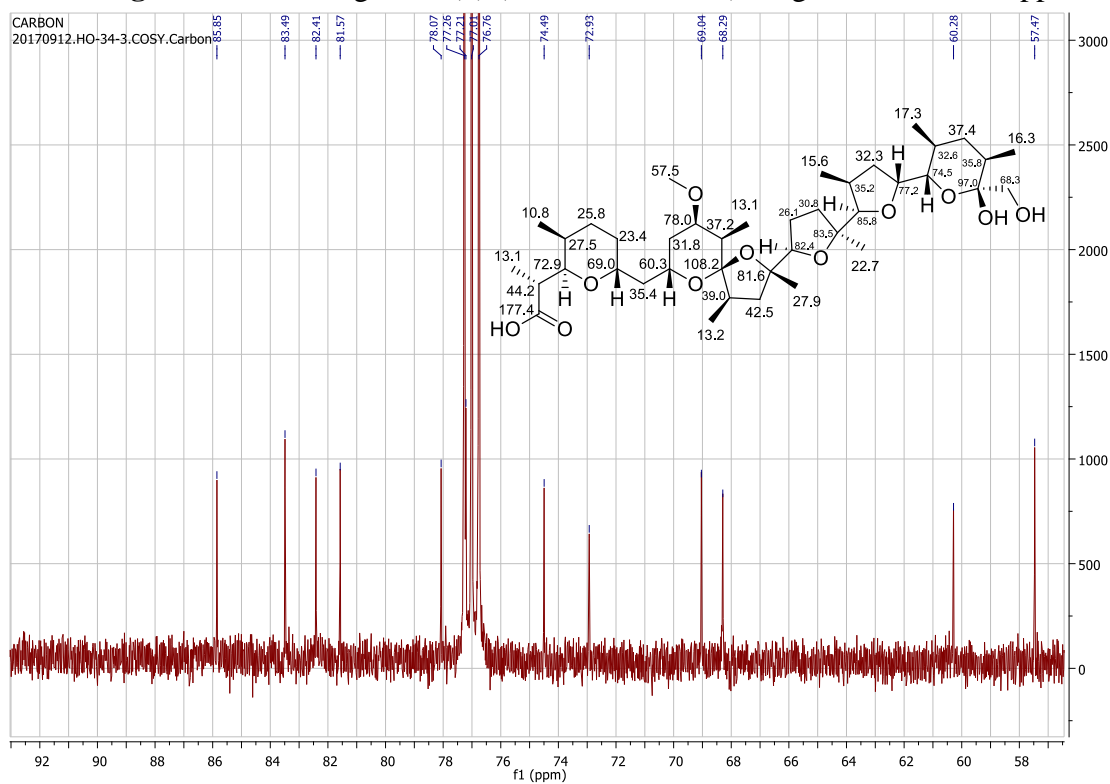

Supplement: S10 Fig — Region of 56 to 110 ppm. (PDF) [file pntd.0007643.s010.pdf]

**S11 Fig. HRESIMS of nigericin (3)**

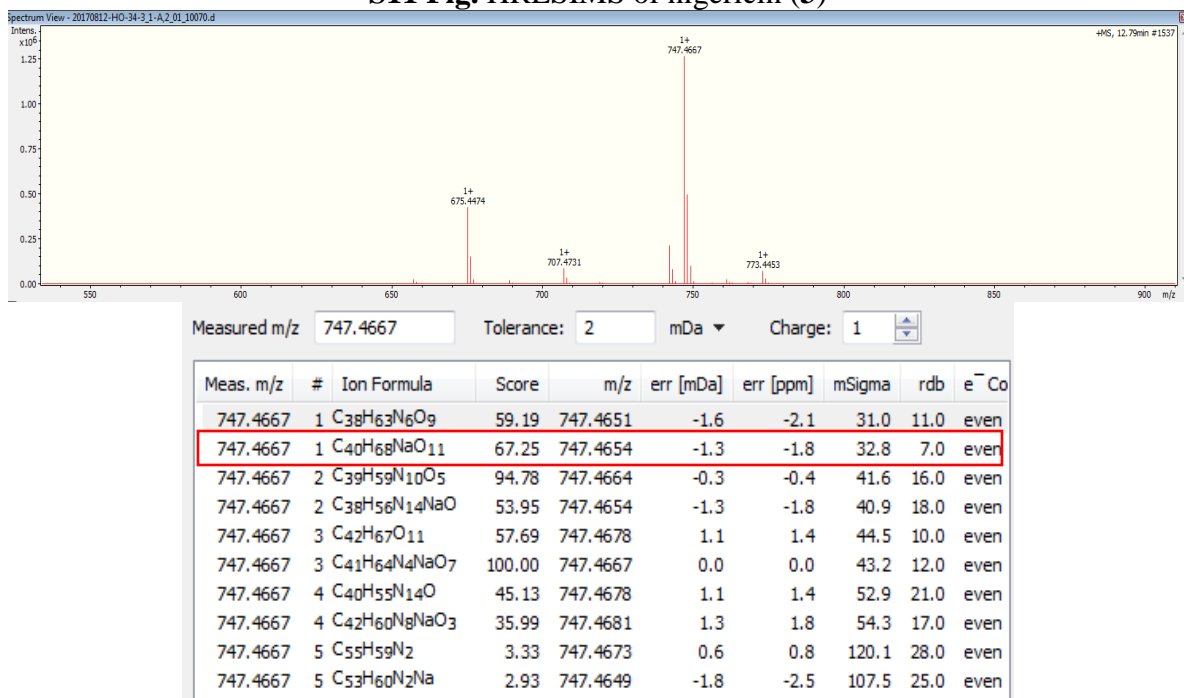

Supplement: S11 Fig — (PDF) [file pntd.0007643.s011.pdf]

**S12 Fig.**  $^1\text{H}$  NMR of dinactin (**4**) ( $\text{CDCl}_3$ , 500 MHz)

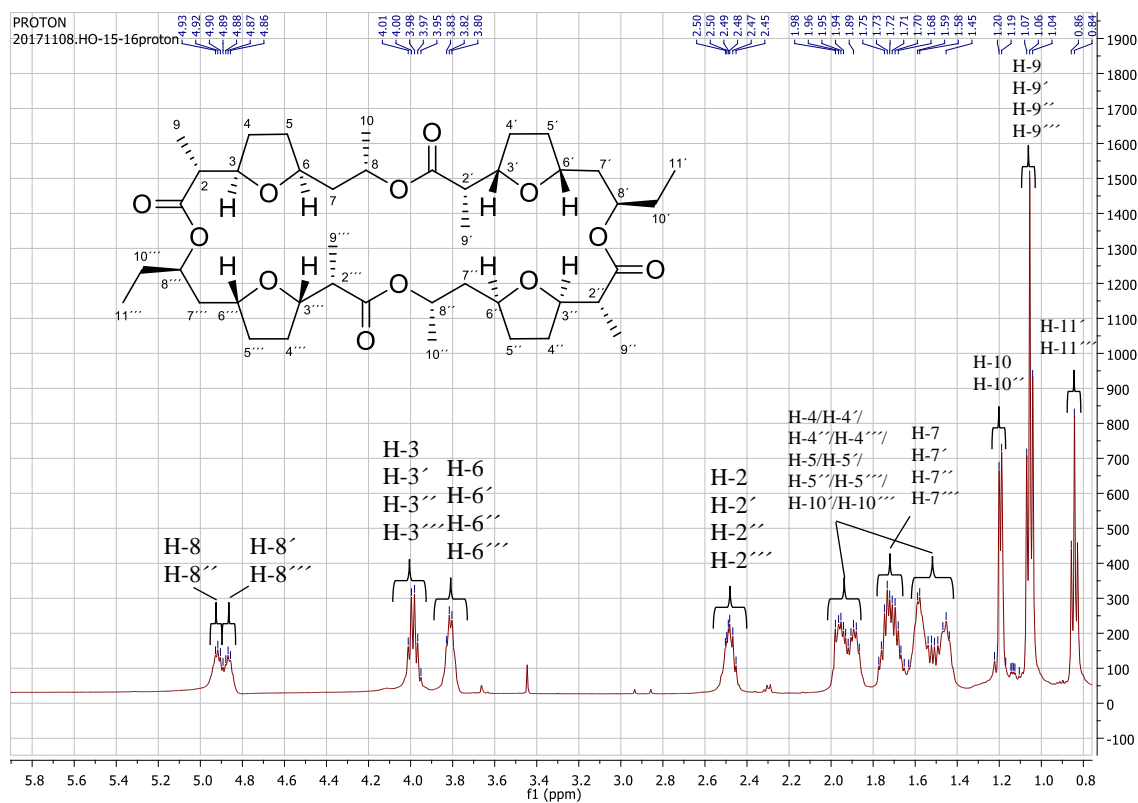

Supplement: S12 Fig — (PDF) [file pntd.0007643.s012.pdf]

**S13 Fig.**  $^{13}\text{C}$  NMR of dinactin (**4**) ( $\text{CDCl}_3$ , 125 MHz)

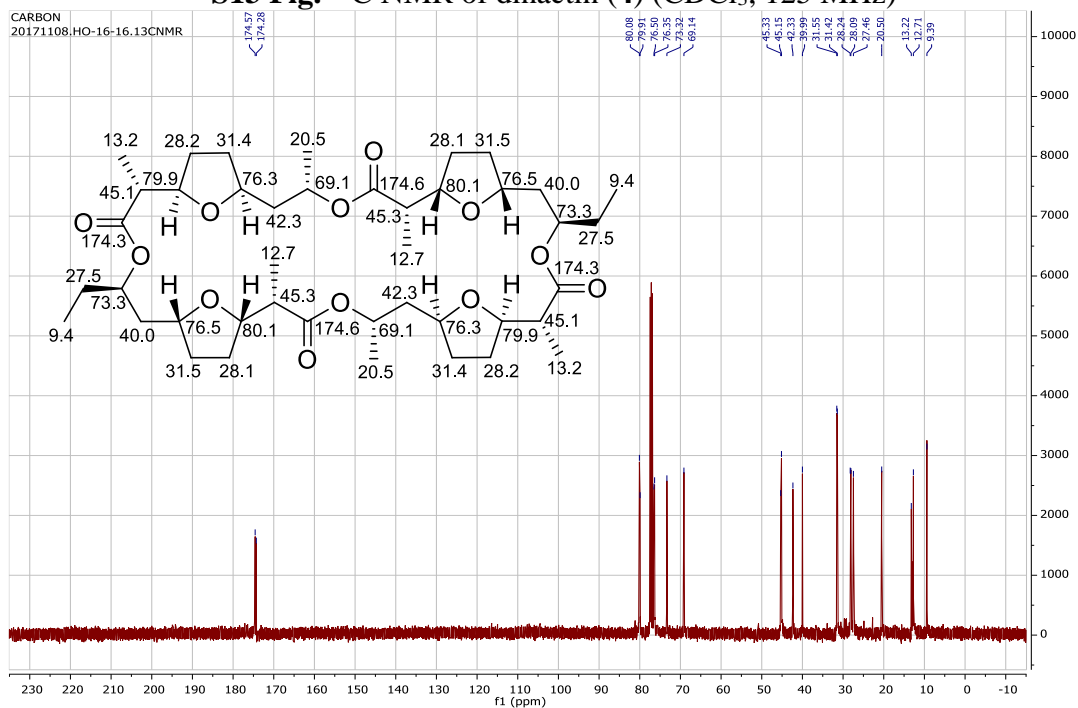

Supplement: S13 Fig — (PDF) [file pntd.0007643.s013.pdf]

**S14 Fig. HRESIMS of dinactin (4)**

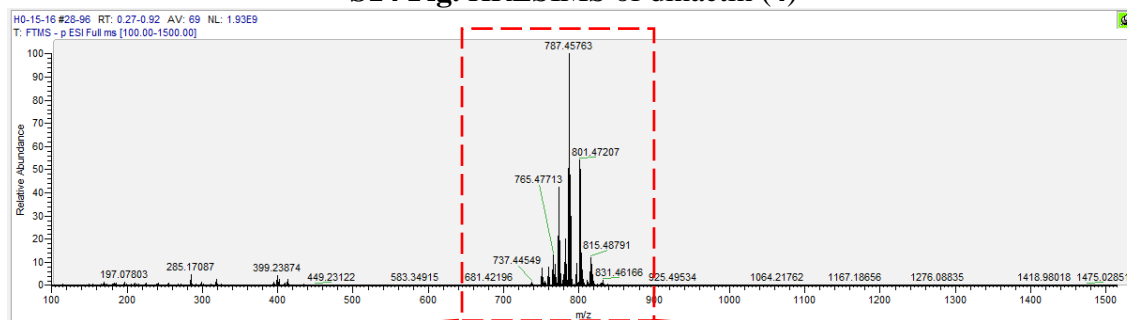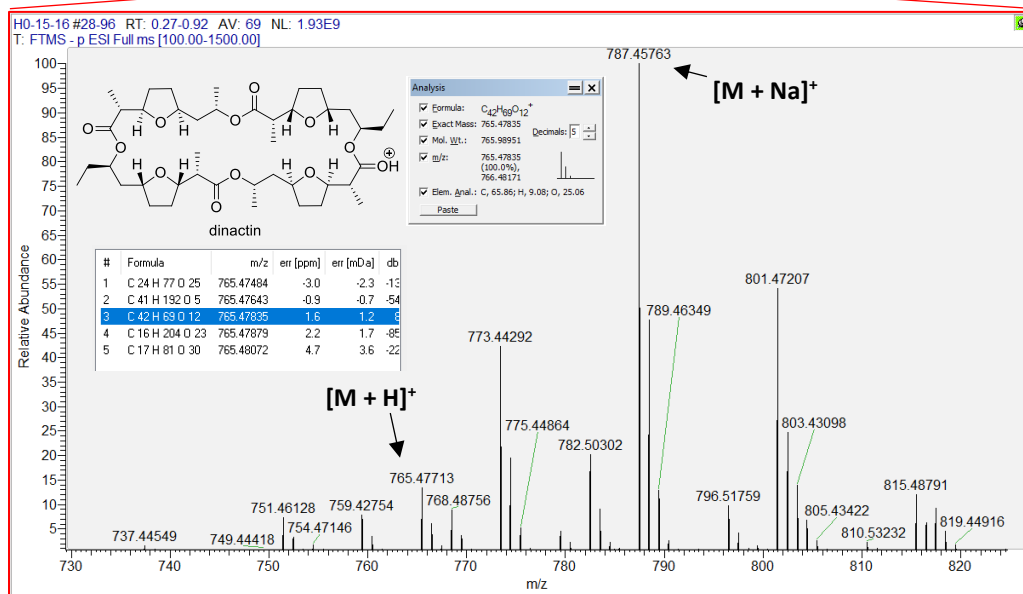

Supplement: S14 Fig — (PDF) [file pntd.0007643.s014.pdf]

**S18 Fig.** LC-HRESIMS of *Atta sexdens* methanolic extract (MRM  $m/z$  386.40, positive mode)

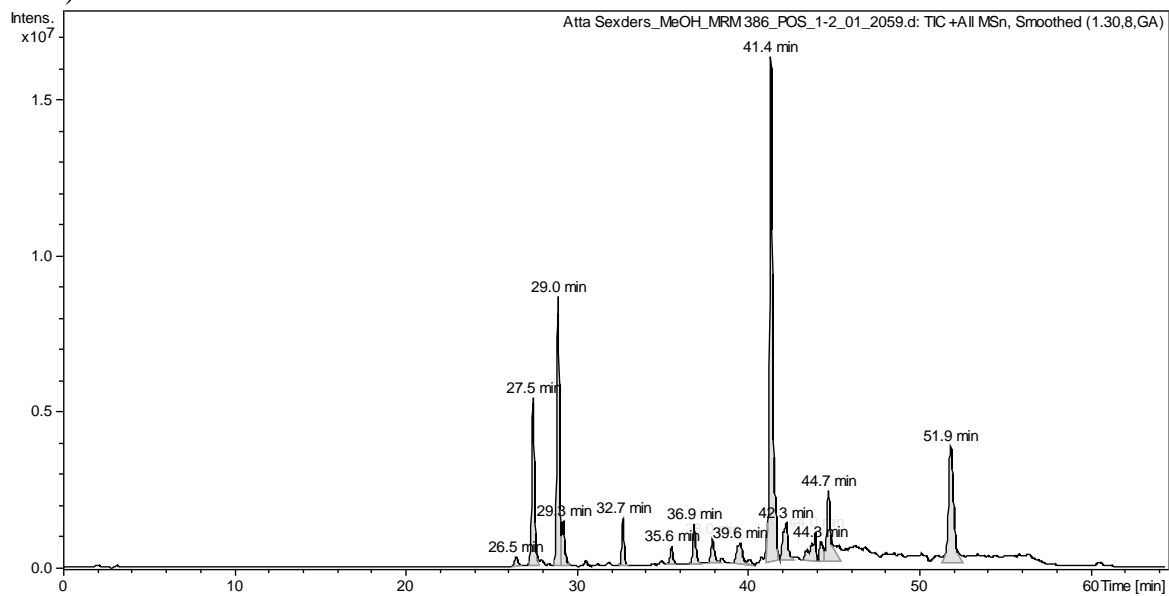

Supplement: S18 Fig — (PDF) [file pntd.0007643.s018.pdf]

**S19 Fig.** MS/MS spectra of compound eluted at 32.7 minutes ( $m/z$  386.40), identified as mer-A2026B (positive mode)

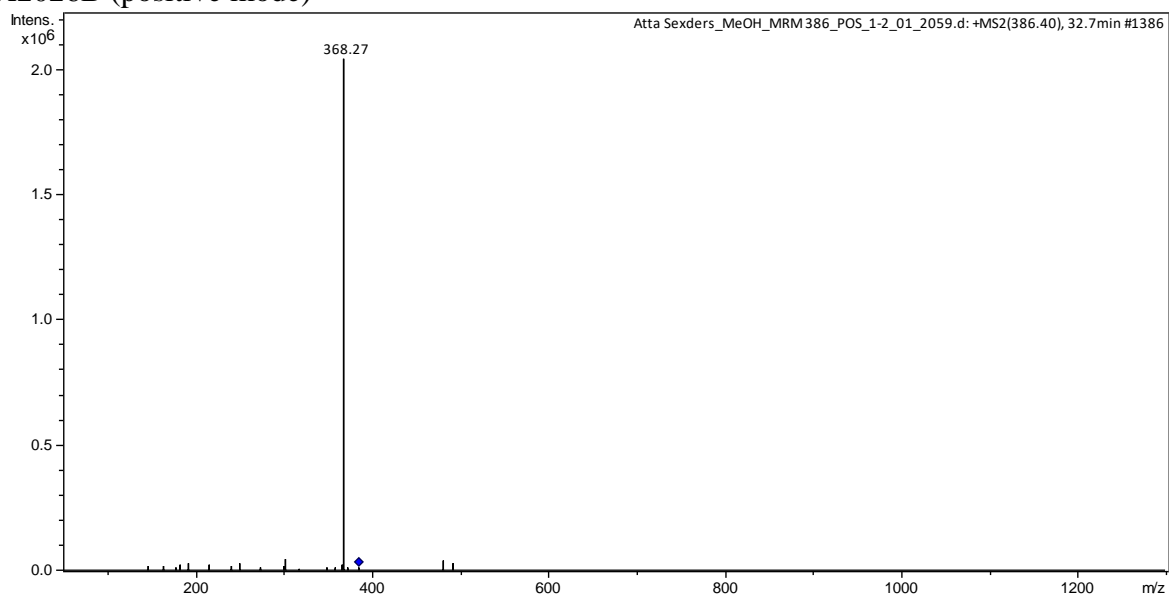

Supplement: S19 Fig — (PDF) [file pntd.0007643.s019.pdf]

**S20 Fig.** MS/MS spectra of mer-A2026B (positive mode)

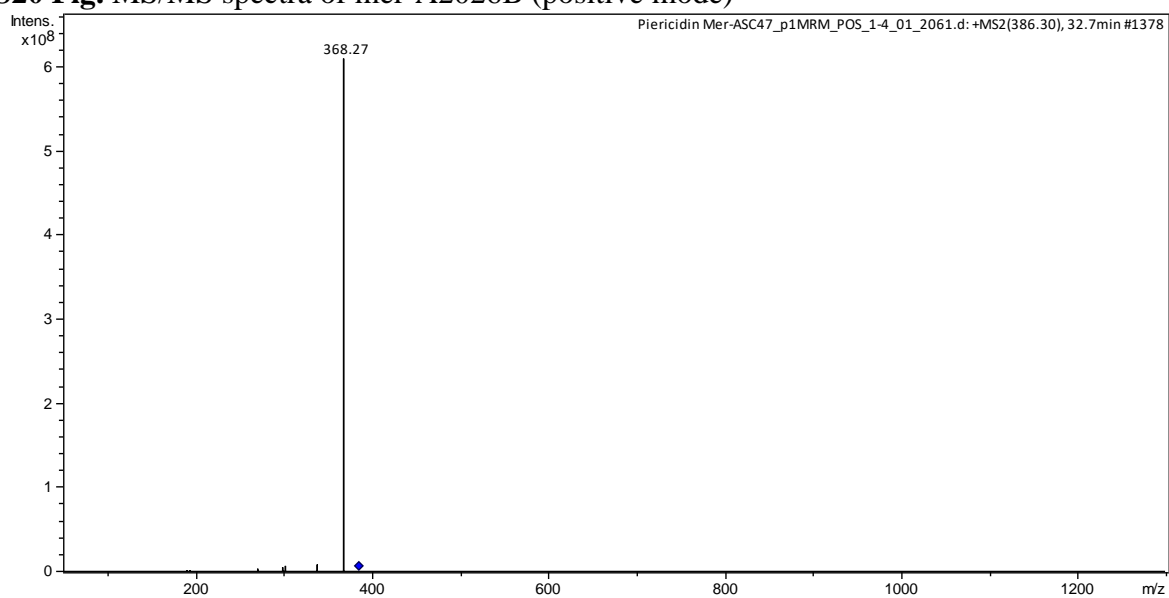

Supplement: S20 Fig — (PDF) [file pntd.0007643.s020.pdf]

**S21 Fig.** LC-HRESIMS of *Atta sexdens* methanolic extract (MRM  $m/z$  416.30, positive mode)

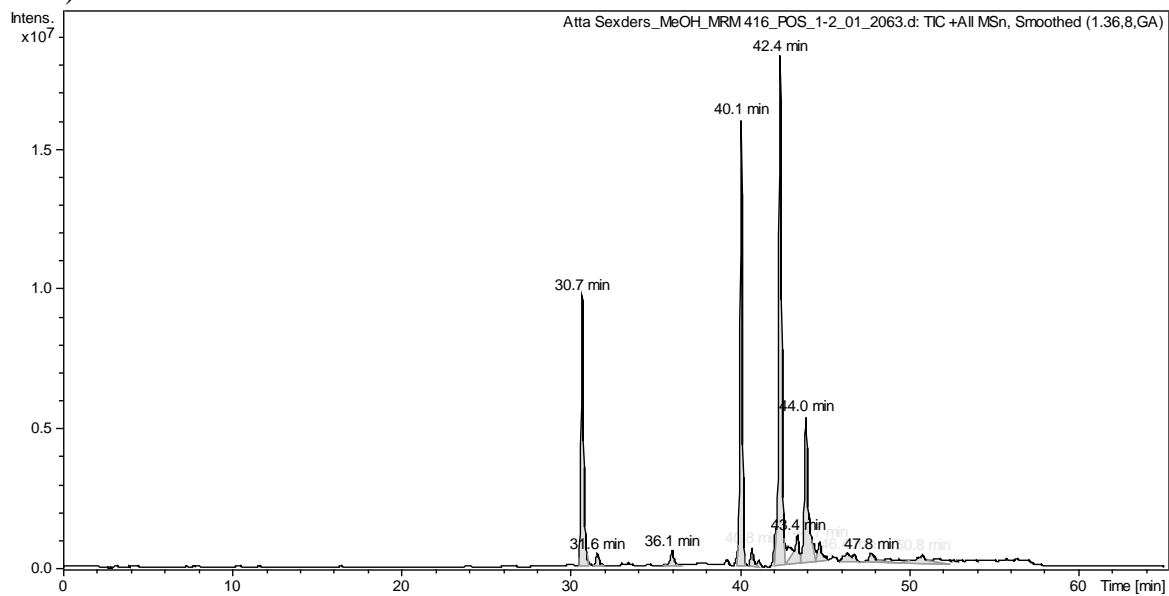

Supplement: S21 Fig — (PDF) [file pntd.0007643.s021.pdf]

**S22 Fig.** MS/MS spectra of compound eluted at 40.1 minutes ( $m/z$  416.30), identified as piericidin A (positive mode)

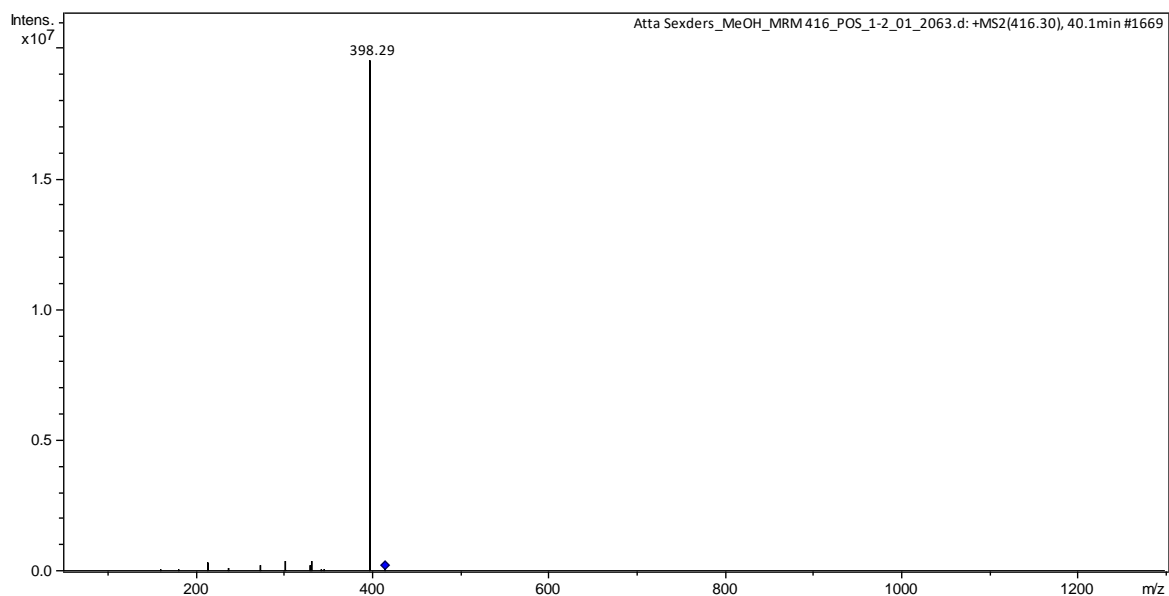

Supplement: S22 Fig — (PDF) [file pntd.0007643.s022.pdf]

**S23 Fig.** MS/MS spectra of piericidin A (positive mode)

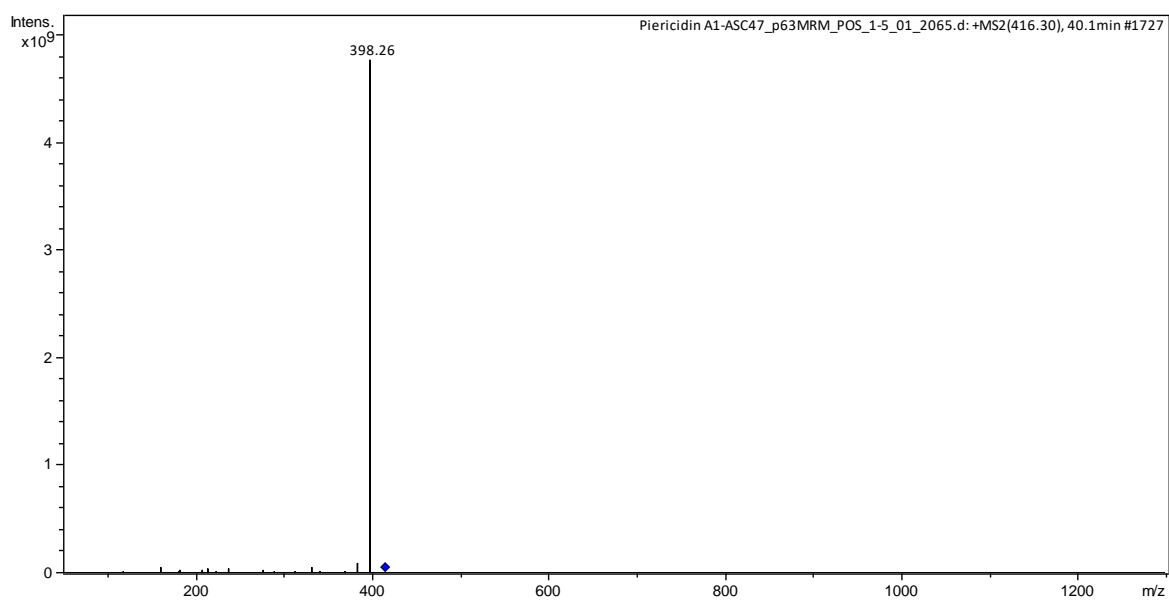

Supplement: S23 Fig — (PDF) [file pntd.0007643.s023.pdf]
