# Supplementary material for: Antifungal compounds from Streptomyces associated with attine ants also inhibit Leishmania donovani
Source: PLoS Negl Trop Dis. 2019 Aug 5;13(8):e0007643. doi: 10.1371/journal.pntd.0007643 (PMC6695191; doi:10.1371/journal.pntd.0007643)
Supplement: S3 Fig — (PDF) [file pntd.0007643.s003.pdf]

S3 Fig. HRESIMS of mer-A2026B (**1**)

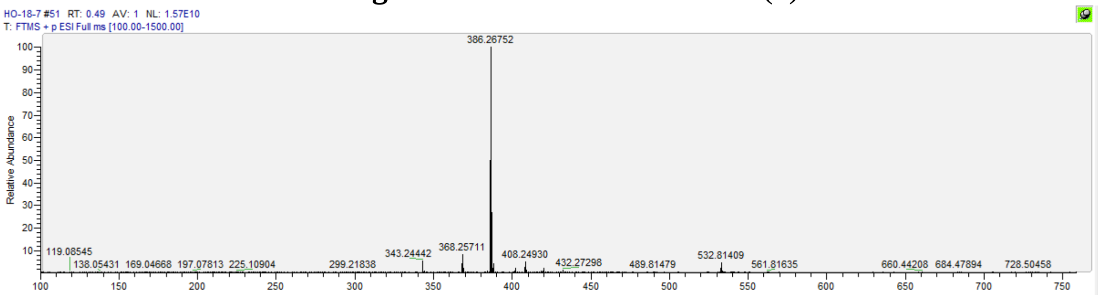

#	Formula	m/z	err [ppm]	err [mDa]	c
1	C 20 H 32 N 7 O	386.26629	-3.2	-1.2	
2	C 21 H 38 O 6	386.26629	-3.2	-1.2	
3	C 5 H 28 N 19 O 2	386.26679	-1.9	-0.7	
4	C 6 H 34 N 12 O 7	386.26679	-1.9	-0.7	
5	C 7 H 40 N 5 O 12	386.26680	-1.9	-0.7	
6	C 22 H 34 N 4 O 2	386.26763	0.3	0.1	
7	C 7 H 30 N 16 O 3	386.26813	1.6	0.6	
8	C 8 H 36 N 9 O 8	386.26814	1.6	0.6	
9	C 9 H 42 N 2 O 13	386.26814	1.6	0.6	-[illegible]
10	C 24 H 36 N O 3	386.26897	3.8	1.5	
