# Supplementary material for: Antifungal compounds from Streptomyces associated with attine ants also inhibit Leishmania donovani
Source: PLoS Negl Trop Dis. 2019 Aug 5;13(8):e0007643. doi: 10.1371/journal.pntd.0007643 (PMC6695191; doi:10.1371/journal.pntd.0007643)

S7 Fig. ^{1}H NMR of nigericin (**3**) ($CDCl_3$, 500 MHz)

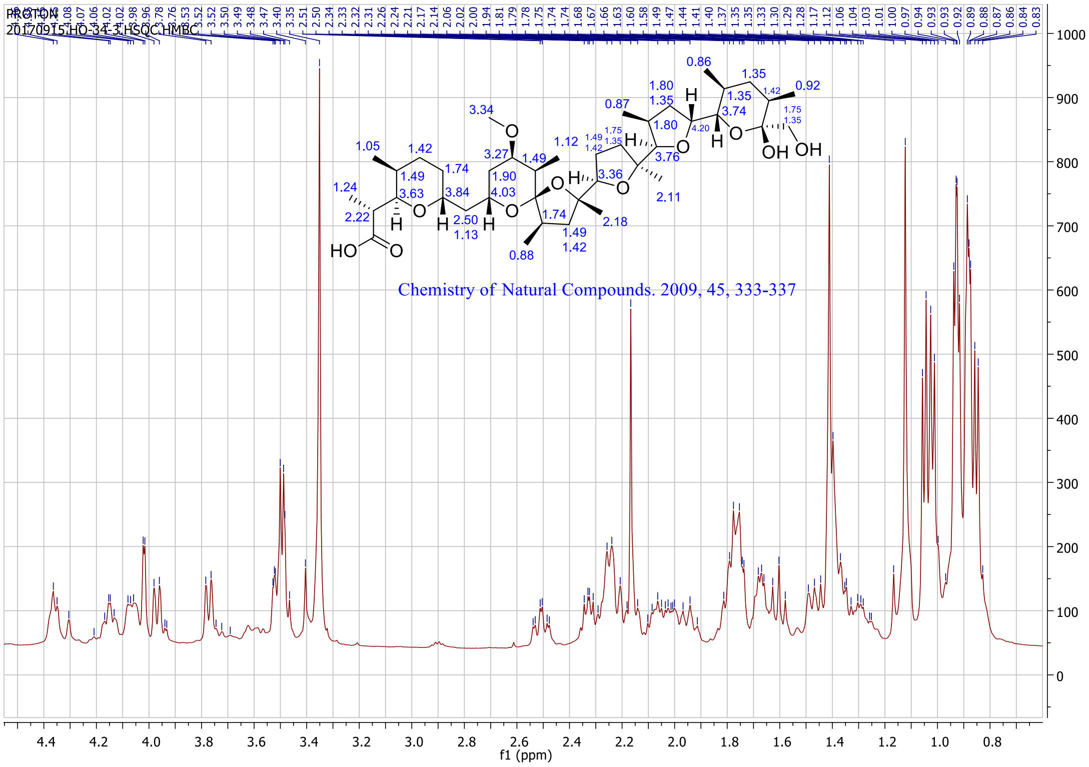

Supplement: S7 Fig — (PDF) [file pntd.0007643.s007.pdf]
