# Supplementary material for: Antifungal compounds from Streptomyces associated with attine ants also inhibit Leishmania donovani
Source: PLoS Negl Trop Dis. 2019 Aug 5;13(8):e0007643. doi: 10.1371/journal.pntd.0007643 (PMC6695191; doi:10.1371/journal.pntd.0007643)

S15 Fig. Antagonist activity of compounds (100 µg) against *Escovopsis* sp ICBG711 (21 days of growth at 28 °C): A) Mer-A2026B (**1**), B) piericidin-A_1 (**2**), C) nigericin (**3**), D) dinactin (**4**), E) Miconazole, and F) Negative control

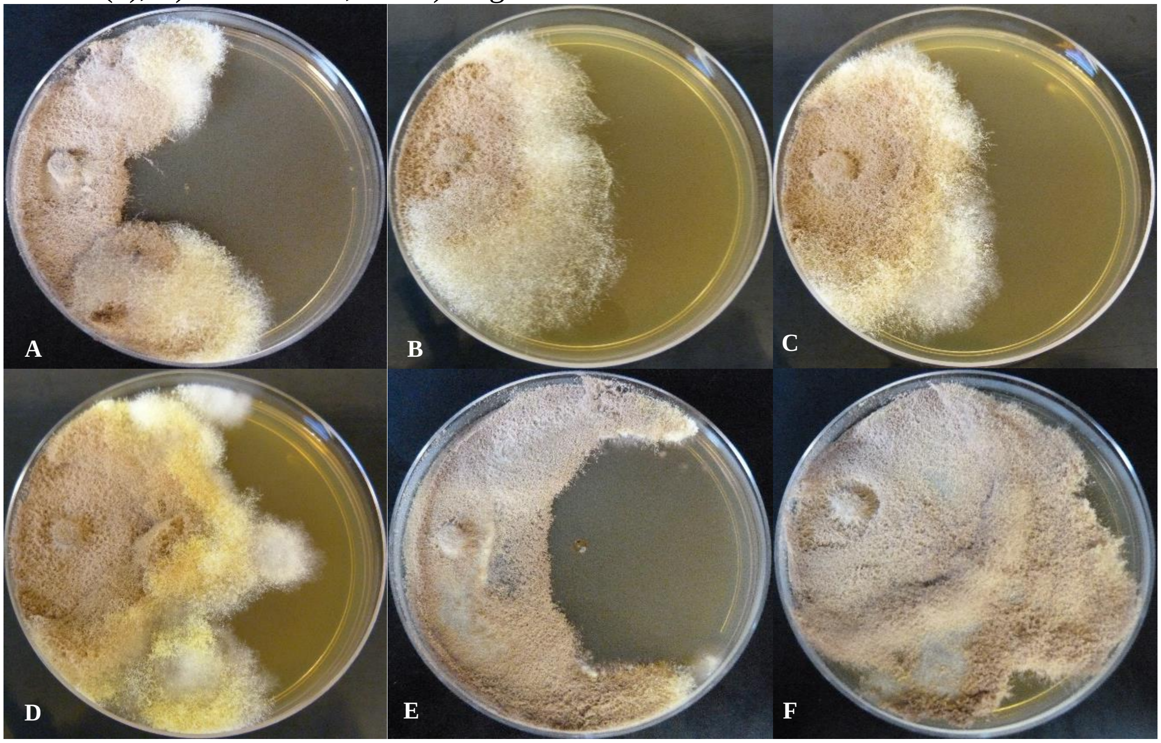

Supplement: S15 Fig — (PDF) [file pntd.0007643.s015.pdf]
